# Italian Community Psychology in the COVID-19 Pandemic: Shared Feelings and Thoughts in the Storytelling of University Students

**DOI:** 10.3389/fpsyg.2021.571257

**Published:** 2021-03-18

**Authors:** Immacolata Di Napoli, Elisa Guidi, Caterina Arcidiacono, Ciro Esposito, Elena Marta, Cinzia Novara, Fortuna Procentese, Andrea Guazzini, Barbara Agueli, Florencia Gonzáles Leone, Patrizia Meringolo, Daniela Marzana

**Affiliations:** ^1^Department of Humanities, University of Naples Federico II, Naples, Italy; ^2^Department of Education, Literatures, Intercultural Studies, Languages and Psychology, University of Florence, Florence, Italy; ^3^Catholic University of the Sacred Heart, Milano, Italy; ^4^Department of Psychology, Educational Science and Human Movement, University of Palermo, Palermo, Italy

**Keywords:** emotional and action connectedness, solidarity, trust, collective mourning, COVID-19, civic-mindedness

## Abstract

This study investigated how young Italian people experienced the period of peak spread of COVID-19 in their country by probing their emotions, thoughts, events, and actions related to interpersonal and community bonds. This approach to the pandemic will highlight social dimensions that characterized contextual interactions from the specific perspective of Community Psychology. The aim was to investigate young people's experiences because they are the most fragile group due to their difficulty staying home and apart from their peers and because they are, at the same time, the most potentially dangerous people due to their urge to gather in groups. The research involved 568 university students, 475 females, and 93 males, with an average age of 21.82 years (SD = 4.836). The collected data were analyzed with the Grounded Theory Methodology, using the Atlas 8.0 software. From the textual data, representative codes were defined and grouped into 10 categories, which reflect the individuals' prosocial attitudes, behaviors, and values. These categories formed three macro-categories, called: “Collective Dimensions,” which includes Connectedness, Solidarity, Italian-ness, Social Problems, and Collective Mourning; “Prosocial Orientation,” which includes Trust and Hope; and “Collective Values,” which includes Values of Freedom, Respect of Social Rules, and Civic-Mindedness. All these macro-categories are indicative of the shared feelings experienced by Italians during the first time of the pandemic. Further practical implications of these results will be discussed, including a consideration of the risk of developing distress and improving well-being, as well as promoting preventive behaviors.

## Introduction

This article will examine the perceived burden of the Covid-19 lockdown on the lives of young people from the specific perspective of Community Psychology.

Lewin (1936) perspective situated this discipline at the boundary between individual and social events (Amerio, 2000; Kagan et al., 2007; Orford, 2008). Therefore, its hallmark is an ecological approach capable of analyzing the interplay of individual, relational, and social experiences (Prilleltensky, 2008; Di Napoli et al., 2019a).

We examined the COVID-19 lockdown considering individual emotions and thoughts as well as actions and significant events of individual life. In our model, we aimed to detect perceptions and representations of individuals in all their emotional and cognitive dimensions as well as the events which influence their experience, together with—for them—significant actions and behaviors (Arcidiacono, 2016). In line with this, we propose a narrative setting, capable of depicting their experience in four individual dimensions (emotions, thoughts, actions, and events). From the ecological perspective, we considered the individual domain of feelings and thoughts, that is, we took a cognitive and emotional perspective. Meanwhile, following Amerio (2000) and Ajzen and Kruglanski (2019), we considered actions as the best individual expression of the interaction with the external world; furthermore, events concern the experiences that occur around people and are the expression of the contexts in which people are immersed.

## The Literature

Lewin stated that “groups come into being not only because of perceived similarity, but because members realize their fates depend on the fate of the group as a whole” (Lewin, 1948; Brown, 1988; Townley, 2017). This certainly also applies to a community. A community can be considered in terms “of the emotional and psychological connections that exist between people and the groups they form and the means by which people communicate the idea of community—it exists through shared meaning” (Kagan et al., 2007, p. 75).

A community is characterized by the presence of some collective dimensions such as emotional connectedness and solidarity, trust, and civic values, and these assume different meanings as circumstances change. It is worth mentioning that Walker (2020) emphasizes the role of we-ness and of the need to create shared ties specifically in times of emergency.

### Emotional Connectedness and Solidarity

In times of crisis and social trauma such as Covid-19, individuals and families change their relationship with the social world and the community.

The journal *Nature* recently published an article on social and behavioral response to Covid-19 asserting that fighting a global pandemic requires large-scale cooperation (Van Bavel et al., 2020). In this pandemic, there are several collectives (for example, family, community, national, and international) which can make decisions to cooperate when faced with such an unexpected social event.

The awareness, fostered by fear (Pulcini, 2002), of being united with other human beings through the perception of vulnerability and weakness leads people to feel a renewed desire for bonding and generates and reinvigorates the desire for community and the need to organize themselves in forms of shared sociality, in other words, the need/desire for coexistence and for a sense of community (Di Maria, 2000; Marta et al., 2016; Procentese and Gatti, under review).

Recent research showed that sense of community is central to a program of protecting citizens' well-being during pandemic conditions (Lie et al., 2020). Also, O'Neill (2004) stated that, as in conditions of disaster, the sense of community favors the protection of communities and that it increases when citizens are taken into consideration by their community (Lau et al., 2008).

The dimensions of the collective refer to the sense of community (SoC) defined as that “feeling that the members feel they belong, to be important to each other and to the group, a shared trust that the needs of the members can be satisfied through commitment to be all together” (McMillan and Chavis, 1986, p. 9).

The four fundamental elements that make up the sense of community are evident in this definition. The first element is the sense of belonging, which refers to a feeling of being part of a community and to the experience of emotional security that derives from this. A second concept involving the sense of belonging is identification with the community, that is, the experience of feeling adequate and well-integrated into it. Finally, the sense of belonging includes the sharing of a system of symbols, which has the main purpose of initially creating and then maintaining the sense of community.

The second element that makes up the sense of community is influence: it is a two-way concept as it is understood both as influence of the community on members and vice versa.

The third fundamental element is the integration and satisfaction of needs, that is, the members' certainty that their needs will be met thanks to belonging to the group since within it there is a sharing of the needs themselves as well as of purposes, beliefs, and values.

Studies have shown that the psychological sense of community is an important component in community initiatives. It is positively related to higher levels of well-being and associated with pro-social behaviors, civic participation, and promotion of social capital (Chavis and Wandersman, 1990; Prezza et al., 1999; Roussi et al., 2006; Pozzi et al., 2014; Ornelas et al., 2019).

In other words, community members who join together will have better chances of satisfying both their personal and their collective needs. The fourth and final element is a shared emotional connection, that is, the presence of strong emotional bonds between the members.

Coexistence is therefore favored by the sense of community that is configured as a catalyst for active, shared, and visible social participation in the entire community of belonging (Chavis and Wandersman, 1990; Hughey et al., 1999; De Piccoli et al., 2004; Christens and Lin, 2014).

Literature has effectively confirmed that SoC is associated with community participation (Florin and Wandersman, 1984; Chavis and Wandersman, 1990; Brodsky, 1996). Both community participation and SoC are interrelated key factors that promote community development and improve the chances that communities will solve problems, enhancing their internal human resources and promoting social empowerment (Talò et al., 2014).

### Trust

In a pandemic situation, social trust and institutional trust are very important issues for overcoming the crisis, as literature has widely demonstrated. Indeed, trust assumes a central role in the acceptance of recommended measures (Paek et al., 2008; Vaughan and Tinker, 2009).

Several studies have examined the role of trust during the H1N1 influenza, highlighting the importance of building public trust for promoting compliance with recommended behaviors (Gilles et al., 2011; Prati et al., 2011; Quinn et al., 2013; Freimuth et al., 2014). Moreover, Van Der Weerd et al. (2011) highlighted the fact that during the influenza pandemic (H1N1), trust in institutions increased, but trust does not always assure adherence to proactive measures. Recently, Sibley et al. (2020) reported that during the COVID-19 pandemic and lockdown, institutional trust and attitudes toward the nation and the government increased, as did trust in science, and trust in the police.

Conversely, in Italy, Stanzani (2020) observed that at the end of the lockdown, institutional trust decreased among Italians, and they only experienced high levels of trust toward activities carried out by NGOs.

This is to say that institutional trust (Lewis and Weigert, 1985; Barbalet, 2009; Luhmann, 2018) expresses judgments about the performance of institutions such as government (e.g., Hetherington, 2005).

Moreover, Rönnerstrand (2016) observed that contextual, generalized trust has been linked to immunization, in line with the literature that argues that being a trustful individual and residing in a community characterized by trust among members influences health and health behavior (Kawachi et al., 1999; Rose, 2000; Hyyppä and Mäki, 2001; Subramanian et al., 2003; Di Napoli et al., 2019b).

Finally, generalized social trust refers to trust toward generalized others who are not directly known (Bjørnskov, 2006; Nannestad, 2008), which occurs when “a community shares a set of moral values in such a way as to create regular expectations of regular and honest behavior” (Fukuyama, 1995, p.153).

Furthermore, a recent study (Imai, 2020) conducted among health workers during the Covid-19 emergency, showed that trust between organizations and workers is essential for improving work motivation and social interaction and cooperation.

### Civic Values

During a pandemic, the sharing of values has a strong impact on social shared identities.

Social values play an important role in addressing the pandemic emergency (Jarynowski et al., 2020), and one's individual perception that others share one's own social values enhances the adherence to norms and behaviors for curbing the spread of the virus (Wolf et al., 2020).

Specifically, Flanagan (2003) and Flanagan et al. (2007) introduced values with respect to civic attitudes: They defined engagement values to explain the position taken and the relative importance attributed by people to issues of a social or political nature. In their view, a constitutive element of civic values is the experience of group membership together with the experience of socialization in one's family and community in general (Sherrod et al., 2002; Marta et al., 2010; Marzana et al., 2012; Alfieri et al., 2014). Wolf (2007) conducted a meta-analysis of civic values and found that civic values are, in order of the most widely studied to the least: political tolerance, understood as the desire to extend civil rights to all, even to groups we do not like; volunteerism, understood as the contribution of one's time to support the activities of community organizations; political knowledge, understood as the awareness of the political system, current events, and political leaders; social capital, as the extent to which a person is networked within their community; civic skills, understood as the experience in and familiarity with activities used to influence the political process; and patriotism, understood as a visceral positive connection to one's country and respect for its national symbols and rituals.

## The Research

Giving voice to people and allowing people to express their needs and desires as well as acquiring awareness about their world are among the most significant goals of community psychology (Rappaport, 1995), rooted in Freire's conscientization 1970 and Martín-Baró's community actions 1994. It should also be recalled that in this discipline, well-being is not only an individual matter; it concerns community interactions and well-being (Di Martino et al., 2018; Di Napoli et al., 2019a). Therefore, social emotional connectedness, community interactions (Prati et al., 2016, 2020), conviviality (Procentese et al., 2019a,b), and participation (Albanesi et al., 2015; Cicognani et al., 2015; Arcidiacono et al., 2016; Churchman et al., 2016; Pozzi et al., 2017) are social pillars for understanding the psychic life. They enrich the merely individual dimensions, compounded by individual emotions and interpersonal relations that act on a family and friendship level.

Thus, at the onset of COVID-19, we decided to explore these dimensions in a group of Italian students. Specifically, we were interested in probing the inner world of young people faced with this unexpected event.

In line with the discipline's vision regarding individual well-being, we investigated how people express these dimensions.

## Materials and Methods

### Aims and Scope

The main goal of this study was to acquire information about the lockdown experience during the pandemic and to understand its meaning and symbolization at individual, local, and national levels.

Framed within the community psychology approach, our interest was to analyze social interactions between individual and social levels during this time. Therefore, we asked our participants to talk freely about emotions, thoughts, events, and actions that they considered significant to share. They were asked to refer to their own personal experience or to feelings and actions related to their relatives, friends, or more generally attributed to this global pandemic.

The aim was to investigate young people's experience because they are the most fragile group due to their difficulty staying home and apart from their peers and because they are, at the same time, the most potentially dangerous people due to their urge to gather in groups. Therefore, storytelling was used as a tool to collect their stories and to probe their meaning and symbolization, developing their reflectivity (Esposito et al., 2017; Salvatore et al., 2018). In fact, in our case, in line with the mission of community psychology to give voice to young people, among the most affected people by the pandemic's social implications, we asked our students to freely express their thoughts and emotions related to their lockdown experience.

Two companion papers will carefully describe their online teaching experience (Novara et al., forthcoming) and their individual feelings and thoughts (Marzana et al., forthcoming; Migliorini et al., 2021).

Here our aim is to probe emotional connectedness and shared actions undertaken by people during the lockdown.

### Participants

The recruitment of the participants took place through the mediation of lecturers in the field of community psychology at five universities in different Italian regions in the north, center, and south. Each instructor invited the students in their own course to participate in the research, filling out an online questionnaire created and distributed through the SurveyMonkey digital platform.

Data collection took place from March 24 to April 1, 2020, i.e., during the week in which the number of cases of COVID-19 contagion reached its peak in Italy.

Participants consisted of 568 university students, 475 females and 93 males, with an average age of 21.82 years (SD = 4.836). For all participant characteristics, see Table 1.

**Table 1:** Characteristics of the participants.

**Age**	**M = 21.82 %**	**SD = 4.836 N (568)**
**Sex**		
Male	16.4	93
Female	83.6	475
**Territorial area**		
North	28.0	159
Center	10.7	61
South	61.3	348
**Sexual orientation**		
Heterosexual	91.0	517
Homosexual	3.0	17
Bisexual	5.3	30
Other sexual orientation	0.7	4
**Housing condition**		
With one or both parents	85.0	483
Alone	2.8	16
With a partner	4.4	25
With one or more roommates	3.3	19
With other family members	4.4	25
**University**		
University of Valle d'Aosta	11.4	65
Università Cattolica del Sacro CCuore Milan	17.3	98
University of Florence	9.9	56
University of Naples Federico II	51.6	293
University of Palermo	9.2	52
Other Italian universities	0.7	4

### Methods and Procedures

Students were asked to describe meaningful events and actions related to their lockdown experience. In a sort of focalized approach (Arcidiacono, 2016), we gave them an open stimulus, but, at the same time, we asked them to delve into specific dimensions: in this case, emotions, thoughts, actions, and events. The text of each single student could be expressed in only a few words, totaling no more than 10,000 characters.

When filling in the form on the SurveyMonkey platform, students were asked to provide informed consent.

Those students were also offered the opportunity to receive individual actions to express social support and the take care of their own student community.

### Data Analysis

The textual material written on the online platform was analyzed by means of the Grounded Theory Methodology (GTM) (Corbin and Strauss, 2008; Charmaz and Belgrave, 2018), using the ATLAS.ti 8.0. Grounded theory, “at the most basic level (…) remains an approach in which researchers use data to develop theory from the bottom up” (Rasmussen et al., 2016, p. 23).

The process of data analysis starts after the first texts provided were analyzed. The aim was for the entire research team to share common meanings to be attributed to the written material. The preliminary coding phase started with a bottom-up approach by coding significant words and sentences. They were then shaped into larger code groups and framed in wider categories.

Several online meetings took place, usually with the participation of 10–17 researchers. A reflexivity-based iterative process was undertaken among all team members. Notes, theoretical memos, and preliminary codes for identifying conceptual categories that shared common meanings were discussed. A reflective procedure (Esposito et al., 2017) was undertaken, in fact, researchers were asked to question themselves to better interpret the findings as they emerged from the texts. The heterogeneity of the research team—particularly in terms of age, professional background, and prior experience with GTM—was used as a resource to better interpret the content and the meaning of the texts. This shared procedure produced several subsequent coding frames, reaching a final shared categorical frame by a consensual strategy. This activity was parallel to the text coding activity that each unit brought to its material. Finally, all codes framed in the shared categories were collected in a common repository (Google Drive folder) and used to start writing the final report. After this, categories, codes and quotations were re-discussed again, and the team reached the definition of the final missing aspects. Last but not least, students of some universities were asked to share in the results.

A preliminary discussion on the collected texts was shared with the students during the course of the study. The preliminary results were also shared with groups of students not directly involved in the research during seminars to which the researcher's team was invited.

A brief report will be provided to all students involved in the research, highlighting the principal results as well as all references to published articles through docent websites and/or Facebook pages.

## Results

The results showed the presence of some sensitizing concepts in the student's storytelling (Blumer, 1969): in other words, “thoughts and hunches” that researchers have as they get started doing research.

**Figure 1:**
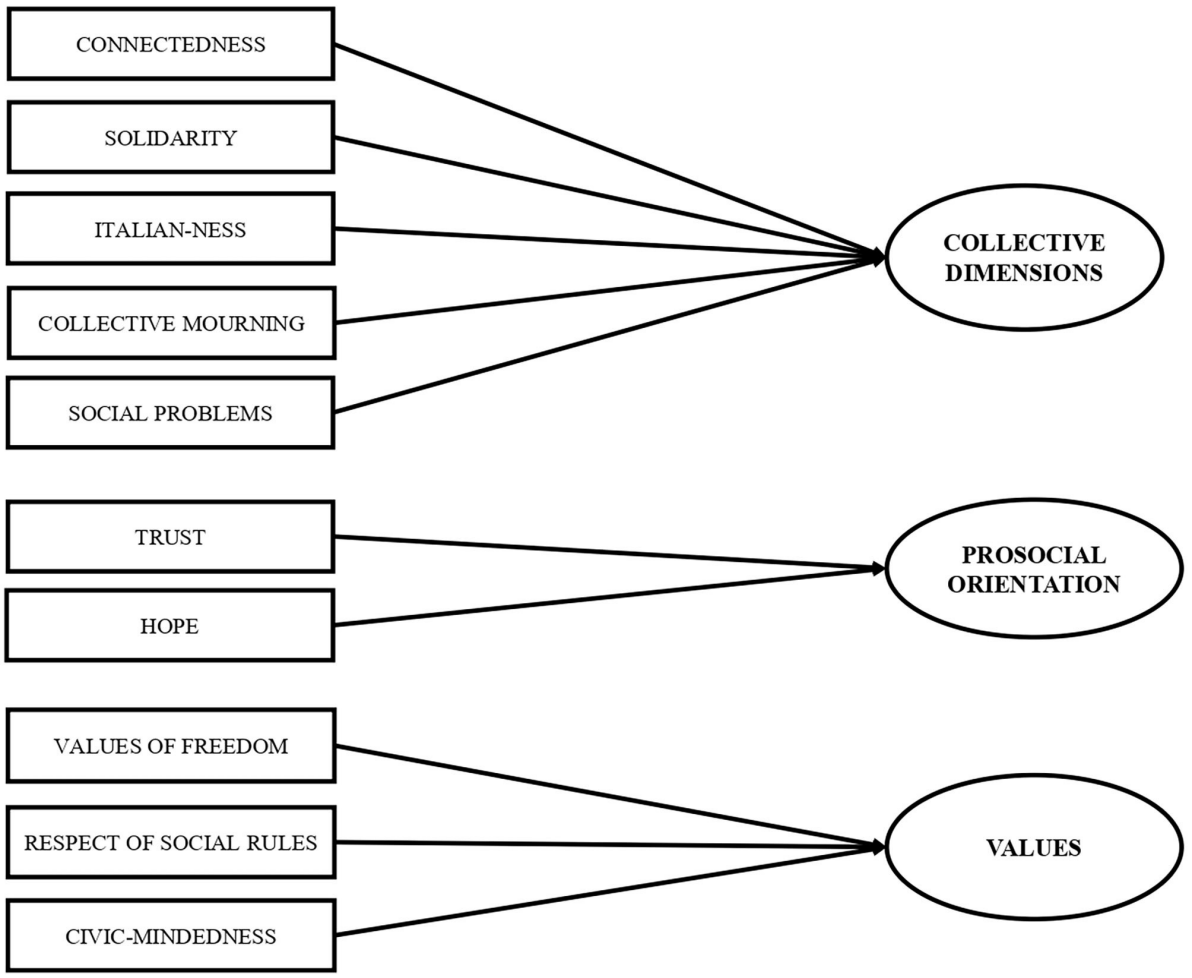
Categories and macrocategories.

The texts were then categorized into collective dimensions such as connectedness, solidarity, social problems, and collective mourning, but the coding also encompassed some specific unexpected thoughts concerning national belonging that were named “Italian-ness” (Figure 1).

The *Collective dimensions* macro-category included several different categories:

Connectedness. A peculiar aspect—consistent with a situation characterized by “*being in the same boat*,” or, better, “*in the same storm*”—refers to sharing the same destiny: the wider community perceives, especially at the peak of the contagion, a close relationship, a “*feeling of unity when facing misfortunes*.” The interdependence of fate, in the lesson learned from Lewin's contributions, is a powerful mechanism in building groups' cohesion. Especially in small local communities or in regions more heavily affected by the infection, such an aspect quickly became the high road toward achieving a great—and, sometimes, unexpected—level of social cohesion.

Other interviewees refer to aspects related to everyday life, such as sharing useful information or exchanging recipes, because cooking was one of the favorite pastimes during the lockdown, with the sense both of expressing one's creativity and of enriching the new ritual of enjoying meals together, at the same table.

In addition, other shared suggestions concerned exercise and workouts due to the discomfort caused by the forced inactivity and the lack of movement perceived particularly in the first days of confinement, and, above all, the most important topic is the sharing of emotions: fear, worry, and uncertainty about the future.

Participants thus wrote about connectedness. They wrote about their own perceptions related to micro- and macro-belonging, and, consequently, about the cohesion experienced in their proximal networks such as their neighborhood, partner, and family: “*the value of having meals all together, around the table, at home.”*

Singing and playing popular songs and hymns on balconies, toasting one another from one side of the street to the other as well as playing traditional bingo (*tombola*) socially together while standing at windows overlooking the same courtyards suddenly became the most relevant ways to express the value of “*being together.”* Shared community emotional connectedness became a new social issue.

At the community level, it is then interesting to note the importance attributed to the *social cohesion* perceived in the local community, “*a great sense of community never perceived before COVID*,” even by the students who were used to spending most of their time outside the home. Social networks were an important way to feel “*far, but, at the same time, close to significant others.”*

Solidarity. A possible outcome of such feelings was the growth of solidarity: “*In my opinion, people may rediscover solidarity, unselfishness, stop thinking about our own interests.”*

Interviewees referred to community solidarity: even if at times it did not involve all the residents, these feelings were widely shared anyway. Solidarity was often defined as “*rediscovered humanity,”* generally referring to the local community, and was also detailed by specifying the more fragile inhabitants: the elderly, people with disabilities or previous illnesses, and those who felt “*lost in an adverse event”* or sometimes “*worthless.”*

Particular attention was paid to healthcare workers and professionals: participants highlighted both the solidarity shown by them toward the whole local community and the inhabitants' appreciation for their work as “*heroes”* in pandemic times.

In addition, similar praise was expressed toward volunteers engaged in providing meals or other basic necessities, or individual protection devices, often lacking in the first phase of lockdown.

Another aspect—emerging with particular emphasis in some contexts—must be underscored: this refers to international solidarity, demonstrated by physicians coming from abroad to help the areas of Italy most seriously affected by the virus or by providing medical devices and financial support. People—and particularly some young people—generally think about other countries as producers of goods or places worth visiting and not as possible helpers.

In “normal” life participants seem to have considered solidarity as a dimension coming from lucky (healthy, medium-high income, and privileged) people toward the most deprived individuals. During the lockdown they experienced the meaning of loss and lack of resources, rediscovering peer-to-peer solidarity.

Italian-ness. The social relationships we experience in daily life also satisfy the need to feel like full members of a community that is territorially recognized and confined in a specific physical and mental space. This feeling of being Italian, and being recognized by others as such, is what we called “Italian-ness.” It includes those anthropological, cultural, and territorial characteristics that connote being Italian. It is not only a question of characters that are objectively unique to a nation, such as a symbol, a geographical border, or a founding myth; a fundamental aspect is, in fact, the common feeling which is that of being part of something bigger and more important that is independent of individual stances and, at the same time, includes them (Reicher and Hopkin, 2001).

What emerges from the research is that the pandemic connected people to the nation—and to some extent to the populace—in a renewed feeling of Italian-ness. Among the symbols, the “tricolor flag” displayed at windows and the “Italian national anthem” sung in unison from the balconies of the houses, or other songs grounded in national memory, all recurred in the texts.

Even the posters displayed on the facades of the houses, with the words “everything will be alright,” had a consoling if not proactive effect. These were the actions through which the population felt less alone or rather, less isolated (probably also from the world) and “closer to all Italian citizens, as never before.”

As one participant wrote: “People who get excited and sing with you in a moment of collective pain can generate reflected joy,” a collective action that outlines an action of “national coping.”

In summary, the pandemic bonded people to the nation in a renewed feeling of “Italian-ness,” highlighting how “in an emergency there are no borders.”

Social Problems. From the social perspective, the description of the problems seems to generate a certain polarity. In some cases, an intrinsically social and collective vision of the problem prevailed: “*. the current situation of many Italian families left without work and in poverty*.;” “*. how complicated the situation is in general*.;” “*. in the elderly in the family, progressively more and more alarming data have generated a strong fear for the future of the country*.;” “*Isolation, necessary for physical health issues, in the long run risks damaging people's mental health*.”

On the other hand, there were many expressions of identification with others, where social concern focused on the individual suffering of the other; therefore, the summative character of the sufferings of the individual emerges as social. In this regard, the texts collected presented a great deal of narrative expressing attention to social problems: “*Yesterday in my city's hospital, an elderly man committed suicide because he had been infected and was afraid of having infected someone else. I'm afraid these episodes will occur again*;” “*This made me think of how many people are now alone and at greater risk*.”

The variety and intensity of this meaning can also be encapsulated entirely within this single statement: “*Anxiety from multiple points of view, for people who are in hospitals and therefore disabled by the virus, for doctors, nurses, healthcare and law enforcement workers, for my grandparents who are elderly, for all those people who unfortunately do not have the means to be safe and for our Italy which has been brought to its knees*.”

Collective Mourning. The Social Mourning dimension seems above all to emphasize the emergence of the community meaning of death, as opposed to the contemporary tendency to consider death as a private event and mourning as a personal elaboration. What is represented in participants' texts, therefore, is not only a fact in itself, but above all the novelty, the surprise, the difference compared to usual living conditions.

In this sense, some aspects appear to be significant. One of these is the frequency with which participants speak about the death of strangers. Fortunately, this is also probably related to a low incidence of family mourning, but the recurrence of this theme underlines the impact at a community level including for the many people not directly involved: “…*seeing all these people die so much that they no longer have room in cemeteries*.;” “. *the pain of all of us sitting on the sofa and hearing the number of deaths every day took away our desire to speak, to smile, people were dying*.;” “*The line of military vehicles leaving the Bergamo hospital*.”

An interesting feature of these statements is that, although they are about strangers, the categories of meaning and the discursive styles of the private dimension of mourning and personal pain are mainly used, as if attuning to the families of the deceased strangers. In the discourse, there is therefore a point of contact between the collective entity of events and the affective and family sphere for the attribution of meaning: “…*he was in a coffin ready to be cremated without family members having the opportunity to say farewell to him and to be celebrated with dignity*.;” “*The impossibility of saying goodbye to the deceased for those who have lost someone*.”

Another element that seems to refer to the community dimension of mourning is the frequency with which the images of the coffins and the line of military vehicles in Bergamo are recalled in the various cities, even distant ones. This refers to a visual representation of experiences conveyed by the mass media. However, this aspect can also be taken for a better definition of the experiences of mourning, because sometimes addressing loss appears in the private dimension, linked to the direct experience of the participants: “*Death of a friend of my mom*;” “*One event that surely struck me was the news of the death caused by the coronavirus of a neighbor of my grandmother's.”* In other cases, the absence of mourning in a social sense and of a community ritual concerning “stranger” mourning, portrayed in the images of military trucks in Bergamo, seems to be emphasized. In this sense, it can be assumed that there is a subtle dissonance between amplified public representations, such as those conveyed by the media, and underestimated individual experiences or direct experiences of mourning.

Finally, in some cases, the speed of contagion transmission number was associated with the Social Mourning macro-category. This occurrence may suggest the profound impression aroused not only by the extent of the losses, but above all by the occurrence, a rampant emergency, coming so fast as to cause anxiety with respect to our practical possibility to combat it.

Collective values. The *Collective values* macro-category includes different categories that refer to behaviors, attitudes, or values related to respect for other people, the rules of living in a community, respect for the rules and responsibility as well as Values of Freedom and civic-mindedness. Civic sense, for example, collects the codes referring to “*staying at home*” as a form of respect for the rules such as adherence to restrictions required by institutions: “*An action that emerges as crucial in this period I believe is respect for the rules and decrees issued. Respecting rules and decrees, being selfless while staying at home*.” Participants, therefore, stated opinions concerning a sense of justice. They referred to the failure of some people to comply with the rules, an injustice toward those who, conversely, did not transgress them. The lack of collective values is connected to this aspect of justice, and it is expressed as individualism, selfishness, and a lack of responsibility.

This refers to an attitude of focusing on oneself and one's needs while ignoring those of others: “*Different people can't help putting their personal needs before collective needs*,” which is reflected in behaviors that denote a lack of respect for the community and negligence in people's behavior.

Some examples of these behaviors are escaping lockdown by train to return home, moving from the so-called “red zones” of Italy, which were considered highly contagious, and irresponsible shopping in supermarket to get as many items as possible. “*The selfishness of these people makes me understand that there is no emotional bond that holds. Faced with the fear of dying these people would also sell their soul to the devil; egoism has never brought benefits to society and to the individual lives of people. I cannot understand how all this can happen without thinking in the least about who is on the other side.”*

An important value on which the interviewees focused was freedom. During this period of lockdown, this assumed the connotation of “*rediscovered value*.” Freedom became a fundamental dimension in daily life, very often taken for granted; it took on great importance when it was taken away from us.

Last, particular attention was paid to the dimension of the environment. Many participants wondered about the effect that the pandemic would have on nature, often speaking of “*the world that is being purified*” and “*planet earth that is reborn*,” coming to the conclusion that “*the negative experience for human beings is becoming positive for nature*.”

### Prosocial Orientation

The *prosocial orientation* macro-category contains two different categories: trust and hope. As far as trust is concerned, the interviewees declared that this state of emergency had increased their confidence and has made them conscious of the importance of trusting their fellow citizens, humanity, and, above all, the public institutions: “*Increasing trust in others is a fundamental premise for moving forward and overcoming the crisis situation*.”

Trust in fellow citizens is basically considered as very important for empowering everybody to adopt precautionary behaviors to protect themselves and other people: “*We really need this type of trust which is trust in others. To believe that people really realize what we are currently experiencing*.”

Moreover, the students refer to an increased confidence in public institutions, above all in the Prime Minister, thanks to the closeness and understanding that the institutions showed toward the citizens: “*Then, leaving aside any political opinion, I've been impressed—positively this time—by the governmental promptness in sending out a message of strength, safety, and trust*.”

Great confidence and recognition were expressed for the Italian lockdown model since it displayed an emphasis on care: “*I felt relieved and a little bit reassured because I had the feeling that the state was putting in place actions and that we as a people were not alone at the mercy of the disease*.”

Trust in the authorities also increased gratitude for the presence of checks on compliance with the lockdown: “*Something positive that I have noticed is the high number of checks that the police are carrying out. In my neighborhood I see police cars passing continuously and stopping passers-by and cars*.”

However, there is a portion of the students who show distrust toward fellow citizens and authorities regarding the management of the emergency. The distrust is mainly toward careless attitudes adopted by other inhabitants and institutions: “*Ignoring the rules imposed by the authorities regarding the coronavirus emergency, it makes me lose esteem and trust in our society*.”

For some of the interviewees, a symbol of hope is represented by the drawings that Italian children made, recognized as symbols of positivity toward the future; children's drawings were also represented as a trigger for serenity, relieving the worries and sadness of the lockdown period.

The saying “*#andràtuttobene*” (#everything will be alright) became a symbol of an individual and collective hope of recovering and returning to normality.

The *hope* category, as an emotional future-directed network, describes what the subjects desired right after the emergency phase.

Hope, however, carries different attributes for the interviewees. Some of the interviewees hope for a revolution compared with what existed before the onset of COVID-19, that is, the possibility of adopting new and different ways of living in the future. Thus, hope for the revolution means importing new ways of establishing interpersonal relationships, taking care of the most vulnerable, having greater care for the environment: “*To rebuild a new a world. More creative. More responsible. More aware. More true. More united. I do not want normality anymore. I want a masterpiece*;” “…*I wish that this moment would bring a real revolution. Inside and outside ourselves. I want to learn the lesson of this hard moment. I do not want to ever lose any bit of what was taught*.”

For some youth, hope will return when they can resume living as in prior everyday life, before the COVID-19 emergency: “*I hope this moment will pass soon*. Panta rei *that is “everything flows” as an important ancient Greek philosopher used to say, so I wish that the stress that our country is suffering will weaken soon*;” “*Hope that everything will end soon is strong*.”

Then some of the interviewees refer to the lack of hope as the inability to imagine themselves in the future. Inability to foresee when the emergency will end makes the interviewees afraid and discouraged.

## Discussion

The coffins left behind in the Bergamo morgue continuously broadcast by media for several days was the image also reported by the frequent dreams (Iorio et al., 2020) that best expressed the dimension of collective mourning; conversely, people talking to each other from balconies was the voice of connectedness. Here visual representations give us the symbolic meaning shared by our respondents. We can, therefore, consider this two-fold image as a core category encompassing shared meaning attributed to this pandemic at the community level. The lines of coffins and people singing and toasting from balconies, respectively express this emotional sharing of collective mourning and the need to express shared feelings of connectedness. In this case, we assumed as a core category not a sentence or a word but two visual images reported by the texts, and it is worth mentioning how visual communication expresses feelings and thoughts (Arcidiacono et al., 2016). At the beginning of the lockdown, this aspiration to share and stay connected was the first spontaneous reaction to the media reports of deaths and infections. Signs of reciprocal solidarity were also expressed and described.

According to Walker (2020), “Psychological research suggests that being in an emergency can create a common identity amongst those affected. Emergencies appear to at least temporarily dissolve social division as the development of this identity facilitates a degree of cooperative altruism even when amongst strangers in life-threatening situations” (p. 4).

Confirming what was reported in the introduction, in the words of the participants, the renewed desire for bonding and community was evident in response to the situation of collective trauma (Ntontis and Rocha, 2020). In fact, unity strengthens the belief in a greater ability to cope with the emergency. It is not trivial to point out that this rediscovered unity also occurred through the recovery of some traditions (social games, cooking, etc.), which represent an anchor to something known, something that does not change (even down through generations) in a time when lives are turned upside down. It is interesting that at a time when the need for freedom and escape is curtailed, tradition retains its consoling meaning for the community, which is to cling onto certainties while adapting them to new needs, even when these are completely unusual and unpredictable.

We have also found this renewed interest in bonds in what we could define as behavioral solidarity toward humanity. In particular, it places the most isolated social categories (such as the elderly, the homeless, etc.) or professional categories less valued by government policies (i.e., doctors, nurses, and the whole health system) at the center of attention and collective sensitivity.

We also found that the epidemic led individual citizens to have a shared emotional experience and to develop a social identity that we have called Italian-ness. In fact, in line with the Intergroup Emotions Theory (Smith and Mackie, 2015), this dramatic event, even if it involved the Italian regions to a different degree, triggered group-based emotions in Italians. These common emotions were independent of the individual level and were linked by a sense of belonging to a common identity. As studies of people exposed to emergencies suggest (Drury et al., 2016, 2019), sharing the crisis situation fostered a sense of belonging among individuals, which was managed to overcome the profound differences that characterize the different regions of the national territory.

This identity was also strengthened by the fact that Italy was at that time the only European country to be severely affected by the epidemic. In fact, during the period in which the data were collected, there was an image, fueled by fear and by social media, of Italians as spreaders of the virus. As postulated again by the Intergroup Emotions Theory (Smith and Mackie, 2015), this discriminatory experience favored social identity thanks to opposition to an outgroup represented by other European countries.

In addition, as Dovidio et al. (2020) argue, the delocalization of the virus that has affected the entire globe and configured itself as a global threat has exported the threat out of the ingroup of belonging, lowering the level of intra-group conflict. This could explain the generalized and newly found trust in the “Other,” which at a higher level of categorization creates a common ingroup identity to which one belongs (Gaertner et al., 2016). In this way, respondents “recognize people are the solution, not the problem” (Jetten et al., 2020, ivi, p. 11). In fact, they become the problem when they do not respect the civic norms that protect the global community, of which everyone—during the pandemic—feels they can be part. The Other, then, can also be a source of mirroring and not just identification (Novara et al., 2019), awakening an empathic capacity that we find in the feeling of collective mourning, in the concern for the economic difficulties of some families, for the risk that workers in the field run, for the community. All these things—as some say—will need to be taken better care of in the future, including reawakening environmental responsibility.

Moreover, in line with literature (Rönnerstrand, 2016; Sibley et al., 2020), there is an increased sense of trust in political institutions in which the interviewees recognize an attitude of care and attention, as well as toward other citizens. Therefore, trust for interviewees is a necessary condition for overcoming the crisis situation.

Finally, it is interesting to note the feeling of hope (Solnit, 2020) in the results regarding not only the prospective image of how we will live together after COVID-19 but also the retrospective image of how we will live in the future based on what has been in the past: conscious collective learning.

Indeed, hope, despite being little explored in its implications in a pandemic situation, is a very important issue, moreover considering it as a socially constructed emotion connected to the quality of life (Scioli, 2007).

This was probably also the benefit of the storytelling method that we used in the research: It allowed the interviewees not only to report their experiences but to narrate them in the psychological sense, accessing an emotional and meaningful elaboration of their experiences, and we know how much this can promote preventive and caring behavior during mass emergencies.

Finally, this study confirms that young people are attentive and sensitive to social issues, as elsewhere described (Alfieri et al., 2019) and have developed a concern for community.

### Limits

This research is not without limits. This study collected experiences of students, and in the classes we included, there was a greater number of females. The number of participants also differs among various universities, but it is quite balanced among the northern, southern, and central regions of Italy. Furthermore, the participants are university students studying in humanities departments; therefore, the sample does not represent all Italian university students and young Italians, more broadly.

In a future study, it would be interesting to develop situated differences among young Italians of different social contexts and to compare the results with youth from other countries facing the pandemic with different strategies.

## Conclusion

This study reveals the initial emotions, thoughts, and actions undertaken at a collective level by students of the North, South, and Center of Italy. Its value is to grasp the primary reaction to the lockdown emergency. It highlights how around Italy, people undertook actions aimed at maintaining continuity among people and overcoming the forced closure of society. For us, as community psychologists, this need for connectedness expresses how community interactions are the pillar of social life.

In this vein, Italian-ness may be understood as a form of shared national identity that makes possible a common identity. Furthermore, the many references to the whole world and environmental needs demonstrate a wider social identification with our planet and all human beings.

Referring to the Elaborated Social Identity Model (ESIM) of crowd behavior developed by Cocking et al. (2009), Carl Walker suggests that it is a “common identity that can result in people helping and supporting each other, even if they are complete strangers. Coronavirus functions in a similar way, positioning groups of people as being under attack from a common and indiscriminate enemy” (2020 p, 4). Therefore, it is not surprising that connectedness actions were among the first collective reactions for our students.

Similarly, solidarity occupied a significant place in collective descriptions of our respondents: “Evidence from a range of different disasters in different countries (Drury et al., 2019) confirms the link between a sense of shared fate and shared social identity, and also between emergent social identity and solidarity” (p. 104).

Narratives coming from the data collected show communities at first astonished and then frightened by the persistence of the contagion. Suddenly, disease and death pervaded everyday life. The psychosocial and collective response, in order to restore an acceptable threshold of well-being—according to Nelson and Prilleltensky (2010) and Prilleltensky (2008)—was to make sense of the whole experience and to undertake all the behaviors needed to care for mental and social health. In the face of social distancing, affecting the opportunities for closer and physical relationships, alternative measures were imagined: virtual connectedness, sense of cohesion expressed by shared rituals (playing, singing, toasting, etc.), organization of community and neighborhood support, first of all, addressed toward the most fragile citizens, such as—for opposite reasons—young people who wished to meet peers and elderly people, heartbroken by the loneliness and by the perceived risk of infection. Creativity advanced in order to provide unusual and effective actions for increasing mutual caregiving, individual and community health and well-being, and sense of community (Chavis and Wandersman, 1990; Talò et al., 2014) in adverse events.

The complexity and depth of these data show the potentiality of storytelling as a tool that offered the students the opportunity to re-think and reprocess traumatic events, a space to rework them. It has proved to be a tool that not only has value in itself but also for the effects it had. Storytelling helped the young people build a meaning into their experiences, to elaborate fears but also to give voice and creative expression to those experiences. Storytelling empowered young people and was a powerful way to improve their overall wellbeing.

From a community psychology perspective, in line with the COVID-19 Statement of EFPA ECPA, 2020, we hope that some collective feelings described by our young Italian respondents during the pandemic will build “new ways of understanding the networks of communities we are part of.” Building trust and solidarity is a long-term process, involving public and private sectors (Capone et al., 2020; Procentese et al., 2020). Communities in many countries are amazingly active in strengthening their feeling of belonging and building new forms of community. Helping to preserve the treasure of engaged, creative, and home-grown ideas and “popup”-solutions will be important if we want to maintain the sense of community and co-creation, which is emerging in our societies.

Therefore, it is to be hoped that these preliminary considerations on the COVID-19 lockdown experience will reach a larger audience and therefore become a lever for social change as the special issue proposed by Castelnuovo et al. (2020) intends to do.

## Data Availability Statement

The raw data supporting the conclusions of this article will be made available by the authors, without undue reservation.

## Ethics Statement

The studies involving human participants were reviewed and approved by University Federico II, Department of Humanities, Ethics Board for Research in Psychology. The patients/participants provided their written informed consent to participate in this study.

## Author Contributions

All authors listed have made a substantial, direct and intellectual contribution to the work, and approved it for publication.

## Conflict of Interest

The authors declare that the research was conducted in the absence of any commercial or financial relationships that could be construed as a potential conflict of interest. The reviewer AMM declared a past co-authorship with one of the authors EM to the handling Editor.
